# Skill Acquisition, Assessment, and Simulation in Minimal Access Surgery: An Evolution of Technical Training in Surgery

**DOI:** 10.7759/cureus.2969

**Published:** 2018-07-12

**Authors:** Ashley Vergis, Sarah Steigerwald

**Affiliations:** 1 Surgery, University of Manitoba, Winnipeg, CAN

**Keywords:** : minimal access surgery, technical skills, evaluation, virtual reality

## Abstract

Diminishing resources and expanding technologies, such as minimal access surgery, have complicated the acquisition and assessment of technical skills in surgical training programs. However, these challenges have been met with both innovation and an evolution in our understanding of how learners develop technical competence and how to better measure it. As these skills continue to grow in breadth and complexity, so too must the surgical education systems’ ability. This literature review examines and describes the pressures placed on surgical education programs and the development of methods to ameliorate them with a focus on surgical simulation.

## Introduction and background

Many pressures have complicated the teaching and assessment of technical skills in postgraduate surgery over the previous decade. A major contributor was the introduction and development of advanced minimal access surgery (MAS). MAS combined with reduced resident work hours, increased costs of operating room time, increased public awareness and the ethics of learning basic skills about patients have further compounded these challenges. These challenges have led to a re-evaluation of how surgical skills are taught and assessed in trainees. Certainly the Halstedian [1] approach to surgical teaching of, “see one, do one, teach one” is no longer favored. This has fueled investigation into the use of skills laboratories and surgical simulation as methods of training and assessing residents. 

The aviation industry has a long history of utilizing simulation for both training and assessment purposes [2-3]. Krummel defined simulation as “a device or exercise that enables the participant to reproduce or represent, under test conditions, phenomena that are likely to occur in actual performance” [4]. Simulation represents a simplified reality that need not include every possible detail or reproduce anatomy with high fidelity (i.e., true to real life) to be effective. Simulated training and assessment environments allow for practice in a realistic setting without the inherent risk of harming others or oneself. This is particularly important when the target performance involves high-stake potentially life-threatening situations, such as with surgery.

Indeed, there are several aspects and evolving challenges that surgical educators must be familiar with in order to facilitate an effective learning environment for technical skills acquisition and assessment in postgraduate surgical education.

## Review

Minimal access surgery

With the introduction of minimal access surgery (MAS) in the 1980s, patients experienced the benefits of smaller incisions, shorter hospital stays, and decreased postoperative pain. These techniques are now common in many surgical specialties such as orthopedics (arthroscopy), thoracics (thoracoscopy), and in general, gynecologic surgery, and urologic surgery (laparoscopy). However, MAS is not without risk and potential serious complications. The skills required to perform this surgery are unique and different than those of traditional ‘open’ surgery. MAS uses optical systems that provide monocular vision. This eliminates depth perception so surgeons must depend on other cues such as light and shading to recreate a ‘stereoscopic’ environment [5]. Additionally, the video image is magnified and projected onto a monitor that is not aligned precisely with the surgical target.  

Minimal access surgery also uses long instruments and trocars; these amplify movements and tremor, and are more difficult to control than traditional instruments. As the trocars are fixed to the body wall, there is a decreased range of motion. Passing instruments through trocars also result in the fulcrum effect, whereby the surgeon’s hand must be moved 180° opposite the direction of the desired movement of the tip of the instrument [6]. The added length of the instruments also significantly dampens tactile sensation. Surgeons rely heavily on their sense of touch to define tissue planes, pathology, and the resistance required to secure knots. Cues such as touch and the interaction of specific instruments with tissue must be learned to help maintain the necessary tactile feedback [6].

The potential for devastating complications combined with the unique skill set required to perform MAS has prompted the surgical community to reconsider the approach to training and assessment of these skills [7]. The need for the demonstration of technical competence by both surgical trainees and practicing surgeons has been highlighted. Societies and regulatory bodies such as the Society of Gastrointestinal and Endoscopic Surgeons (SAGES) and the European Association of Endoscopic Surgeons (EAES) stipulate minimum requirements for those learning MAS with an emphasis on training both in the operating theatre and in the skills courses [7-9].           

Work hour restrictions

In 2003, the Accreditation Council for Graduate Medical Education (ACGME), the professional body responsible for accrediting residency-training programs in the USA, implemented an 80-hour resident workweek restriction [10]. In Canada, while there are currently no pan-Canadian limitations on resident duty hours, there are restrictions within each province. Manitoba has a weekly duty hour limit of 89 hours per week. In Quebec, residents’ duty hours are limited to 72 hours per week with a limit of 16 consecutive hours per day [11]. This may be implemented in other jurisdictions in the next few years. These reduced hours combined with the challenging skill set afforded to MAS, make training of skills and demonstration of competency outside of the operating room even more important.

Laboratory simulation-based training and assessment

Laboratory simulation-based skills training and assessment for MAS surgical skills include lower fidelity physical box/video trainers and higher fidelity virtual reality trainers. The physical box trainers use actual laparoscopic instruments and an optical system that allows the trainee to perform tasks under videoscopic guidance [3]. Virtual reality trainers use computer-generated instruments to perform computer-generated tasks. There is a spectrum of machines available now with many of them incorporating haptic feedback.

Significant research has been performed in studying the validity and reliability of simulation-based training for MAS skills over the last two decades. Training in lab-based settings results in improvement of skills both in the simulation setting and in the operating room. Simulation is now established as an effective method of skill acquisition [12-23]. 

Video trainers have been incorporated into the assessment of MAS skills via the fundamentals of laparoscopic surgery (FLS) program. FLS is a simulation-based assessment and certification program developed by SAGES [24]. It has been thoroughly validated [5, 12, 24-27] and is a requirement to be eligible for certification by the American Board of Surgery [24, 28]

Virtual simulators have also been shown to be capable of assessing the psychomotor skills necessary for MAS [19, 29-30], although there is less research in this area than with video trainers.

Current assessment of technical skills in surgical programs

A trainee’s technical skills are constantly evaluated throughout residency training [31]. This is most often a subjective assessment performed by practicing staff surgeons as a resident rotates through a particular surgical service [27]. At periodic intervals throughout the rotation, the staff surgeon fills out an in-training evaluation report (ITER) that is used to assess all areas of the trainee’s surgical competence. The ITER is composed of a list of rating scales with sections such as knowledge, professionalism, technical skill, teamwork, and communication skills that the evaluator completes to evaluate the candidate’s clinical competency [3]. The purpose of the ITER is to provide an accurate assessment of the trainee’s performance abilities and useful feedback to both the trainee and the program [32].

The ITER has some advantages as an assessment tool. It can encompass aspects of competency such as professionalism, teamwork, and communication skills that may not otherwise be evaluated. It is inexpensive and no special equipment is required. In addition it allows for multiple observations over time [3].

However, there are criticisms of the ITER as a way to evaluate technical skills. ITERs have been found to have poor validity and limited inter- and intra-rater reliability [3, 31-32]. According to Turnbull et al. this is primarily due to two major factors [32]. First, faculty evaluators often do not receive training in the evaluation process and correct use of the ITER, which results in a number of rater errors. Errors of distribution include a range restriction or central tendency error and a leniency/severity or dove/hawk error. In central tendency errors, evaluators use the mid-range average values and fail to use the whole scale, while in leniency/severity error there is a tendency to assign predominantly low or high scores [33]. Additionally, ITERS may be subject to the halo effect, a correlation error that commonly occurs where the raters’ overall impression of the trainee influences the assessment of each individual component of the evaluation [33]. Second, ITERs are not usually filled out on a daily basis, but rather in a retrospective fashion at the middle or end point of a rotation. Consequently, they are based on a recall of events and are generally not sufficiently detailed or accurate.

Procedural logs record the number and type of procedure that a resident completes throughout their training and are often used as a marker of technical competence [31]. These are a requirement for specialty certification by the American Board of Surgery, where an applicant must demonstrate their participation in a minimum number of cases in the various areas of general surgery [3]. A problem with this is that the number of cases required to achieve competency can vary substantially between individuals and participation in a procedure does not ensure competency [34]. In addition, the reliability of the data recorded can potentially be questionable. Trainees are often in a rush while logging procedures, or do so retrospectively at a time remote from the operation.

Currently, many surgical programs use a combination of ITERs and procedural logs to assess whether or not a trainee’s technical skills are sufficient to progress to the next level in their training.

In-training evaluation reports are summative as opposed to formative measures of assessment. They do not provide residents with a concrete understanding of the particular aspects of their technical skills that need improvement [35]. On the other hand, global rating scales are reliable, have high inter-rater reliability and construct validity [36-37]. Residency programs across the country are trying to incorporate the use of these instruments into the evaluation of their trainee’s technical skills. However, this does not appear to be done routinely in the majority of programs.

Validated instruments available for evaluation of surgical technical skills

Objective Structured Assessment of Technical Skills

Martin et al. developed objective structured assessment of technical skills (OSATS) in 1997 as a novel way to more objectively evaluate the technical skills of surgical residents [38]. It is a performance-based examination designed to assess the technical competency of surgical residents that consists of eight 15-minute bench model stations. Each model simulates portions of relevant operative procedures. Examples of these stations include closure of a skin incision, insertion of a T-tube, hand-sewn bowel anastomosis, and control of an inferior vena caval hemorrhage [39]. Two or more observers evaluate each task using a global rating scale with seven dimensions that are each related to some aspect of operative performance. The dimensions assessed include respect for tissue, time and motion, instrument handling, knowledge of instruments, use of assistants, flow of operation, and forward planning and knowledge of specific procedure [38]. Each dimension is graded on a five-point scale with the middle and extreme points anchored by specific descriptors to help clarify scoring [39]. There is also an overall pass/fail judgment for the examiner to make based on their perception of overall performance [38]. In this original study and a subsequent follow up of larger size, the global rating scale was compared to task specific checklists. Both studies demonstrated stronger construct validity and greater reliability for the global rating scale compared to the checklists, and the checklists are no longer used as part of the OSATS scoring assessment.

**Figure 1:**
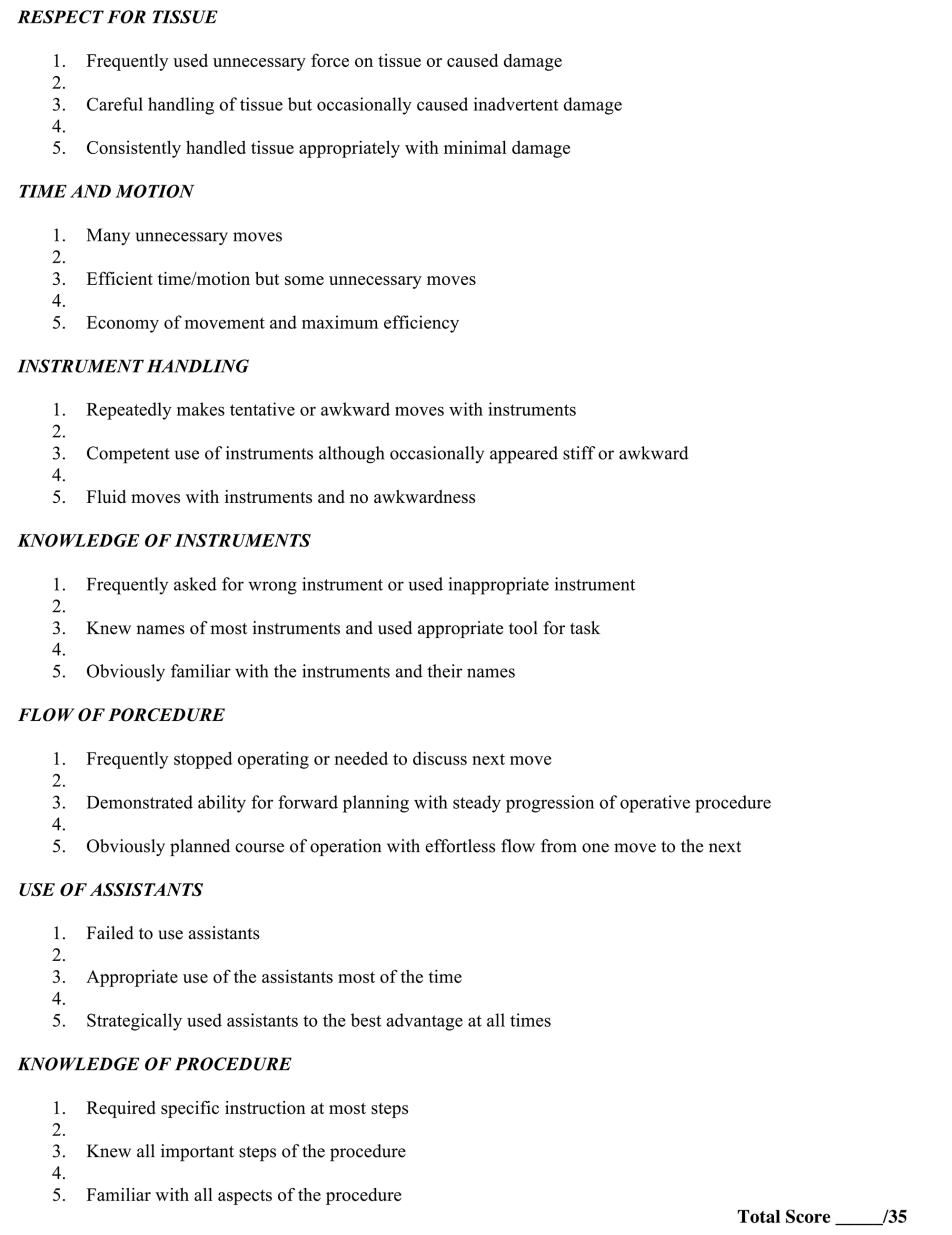
Objective structured assessment of technical skills (OSATS).

The OSATS (Figure 1) has been extensively validated and shown to be an effective method for assessment of surgical skills [40]. It does, however, require significant resources and time on the part of faculty evaluators [31]. Over the past 15 years, it has moved from use in the laboratory setting to use in the operating theatre [21, 36-37]. OSATS is validated and used for the evaluation of both open and laparoscopic surgical skills. It is viewed as the standard for skills assessment [39].

Global Operative Assessment of Laparoscopic Skills

Originally developed by Vassiliou et al., global operative assessment of laparoscopic skills (GOALS) is a global assessment tool used to assess intraoperative skills in laparoscopy [35]. It is composed of a five-item global rating scale and two visual analog scales (VAS) for overall competence and case difficulty. The five domains examined are depth perception, bimanual dexterity, efficiency, tissue handling, and autonomy [35]. Each domain is scored on a five-point scale for dissection of the vesicular bed during laparoscopic cholecystectomy, with the middle and extreme points anchored by specific descriptors to help clarify scoring [41]. The two VAS are 10 cm lines with descriptive anchors at each end. The first VAS measures how technically difficult the operation is, and the second measures the operator’s overall competence. The evaluator marks the appropriate spot on the VAS with an X [35]. 

Vassiliou et al. demonstrated construct validity not only for the overall score, but also for each of the five GOALS items individually [35]. Excellent internal consistency and inter-rater reliability were also demonstrated for the five-item global rating scale. Although the VAS did demonstrate construct validity, inter-rater reliability was weak and we feel that it does not add benefit to the five-item global rating scale. It is still included at the bottom of the rating scale; however, most studies published since the original in 2004 do not utilize the VAS.

Fried et al. further validated GOALS as an assessment tool by demonstrating that it has concurrent validity by comparing GOALS scores with assessment of skills using a simulator [12]. In 2007, Gumbs et al. demonstrated construct validity for GOALS for assessment of a complete laparoscopic cholecystectomy and laparoscopic appendectomy [41]. This has added further evidence to the value and effectiveness of GOALS as a global assessment tool for laparoscopic skills.

**Figure 2:**
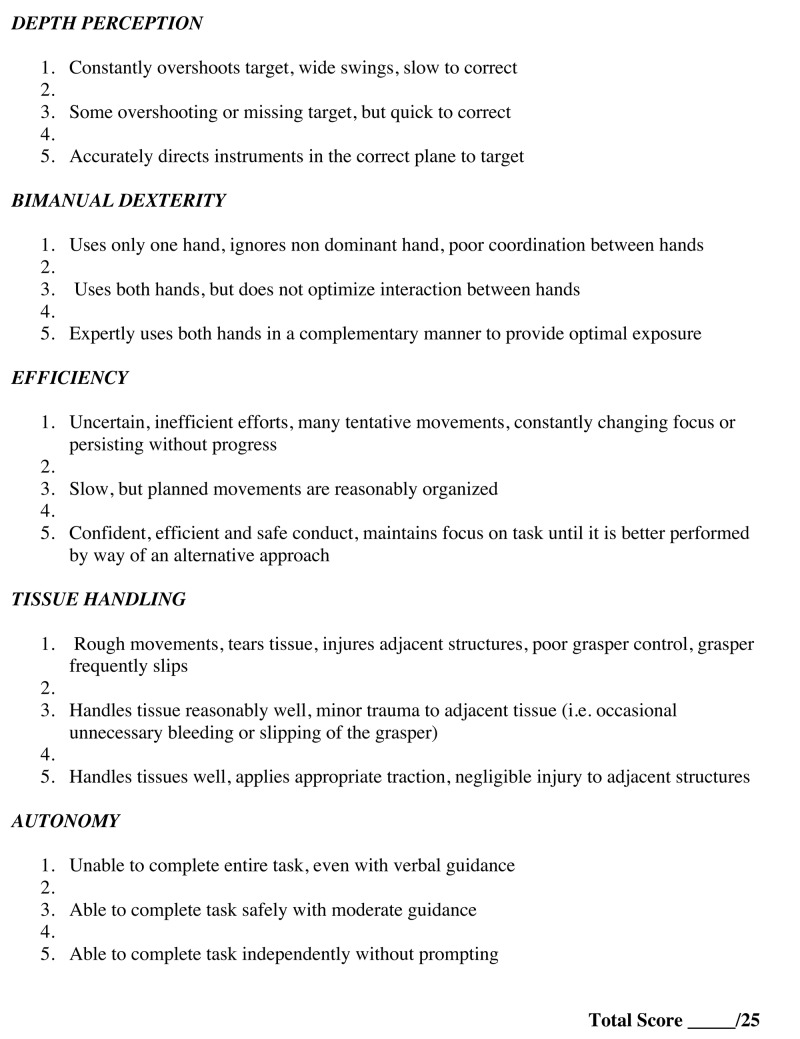
Global operative assessment of laparoscopic skills (GOALS).

To date GOALS is the only global rating scale that has been developed and validated specifically for the assessment of laparoscopic surgery (Figure 2).

McGill Inanimate System for Training and Evaluation of Laparoscopic Skills

In 1998, Derossis et al. set about developing a method to quantitatively assess technical skills in laparoscopic surgery by measuring performance through a series of exercises performed on a surgical simulator [5]. The result was the McGill inanimate system for training and evaluation of laparoscopic skills (MISTELS) simulation system. The original program consisted of seven standardized tasks performed in a trainer box under videoscopic guidance. The tasks were derived by a review of videotapes of basic and advanced laparoscopic procedures by expert laparoscopic surgeons. The face and content validity of the tasks was confirmed through questioning a large group of 44 expert laparoscopic surgeons [12]. The original seven tasks ranged in difficulty and the skills they aimed at developing. Some of the exercises were chosen to develop basic dexterity skills, whereas others emphasized the use of specialized instruments and particular laparoscopic techniques. The tasks consisted of peg transfer, pattern cutting, clip application, placement of a ligating loop, mesh placement over a defect and intracorporeal and extracorporeal suturing [5]. Since the original publication by Derossis et al., the clipping and mesh placement tasks have been eliminated secondary to a lack of validity and limitations in feasibility and cost [3].

Performance of each task is graded objectively, taking into account both precision of performance and speed. The score for an individual task is calculated by subtracting the time required to complete the task, in seconds, from a preset cutoff time [27]. If the time to complete the task surpassed the preset cutoff time, a score of 0 was assigned. A penalty score for inaccuracy is also deducted according to a predetermined system [5].

The MISTELS system has been further validated since the original study and has been shown to be a highly reliable and valid system [3, 6, 12, 18, 27]. In 2004, Fried et al. demonstrated predictive validity of the MISTELS model by showing that its scores correlate highly with intraoperative measurements of technical skill in laparoscopy (*r* = 0.81; p < 0.001) [12]. For this, they evaluated a laparoscopic cholecystectomy using a modified version of Vassiliou et al.’s GOALS rating scale [12]. The SAGES have made MISTELS the manual skills component of its FLS program. These skills are the current gold standard for the assessment of laparoscopic skills outside of the operating room. 

Fundamentals of Laparoscopic Surgery

Fundamentals of laparoscopic surgery is an educational program developed by SAGES. This program was launched in 2004 [25]. The overall goal of the FLS program is to teach and assess the basic cognitive and psychomotor skills required to competently perform laparoscopic surgery [25]. 

The FLS program consists of a didactic module and manual skills training practicum for education, and an examination component to assess competency. The cognitive portion of the educational program addresses four broad content areas: preoperative, intraoperative, and postoperative considerations and basic laparoscopic procedures [26]. The manual skills training portion of the program is based on the MISTELS program originally developed by Derossis et al. at the McGill University. Competence is assessed through a two-part examination, consisting of a cognitive computer based test and a proctored technical skills component.

The FLS program has been thoroughly tested and validated as a teaching and assessment tool for laparoscopic skills [5, 12, 24-27].  It meets the standards for high stakes examination. As of 2010, the American Board of Surgery requires its candidates to be FLS certified [24, 28].

Imperial College Surgical Assessment Device

The Imperial College surgical assessment device (ICSAD) is another method of assessing surgical skills that utilize hand motion analysis.  Sensors are placed on the dorsal surface of the trainee’s hands while they perform a task and the hand motion is tracked. Their movement is translated into a computerized tracing of hand motion by the sensors. This provides an index of technical skill in both laparoscopic and open procedures that has been shown to have good concordance with OSATS scores [42-43]. Although the ICSAD has shown to be a valid indicator of operative skill, the evaluation metrics that are produced do not provide the trainee with feedback that can be used effectively for educational purposes [12, 31].

Potential models available for surgical skills assessment

Patients

While there has been a move away from teaching basic skills in the operating theatre, patients remain an integral part of surgical training. An operation can be reduced into its component parts that can be learned and mastered over a number of operations on separate patients. This is best done under the direct guidance and supervision of a staff surgeon [44]. However, there are a multitude of ethical considerations when allowing trainees to operate on patients. This, combined with fiscal restraints, work hour reductions and restricted operating room time, make it important to evaluate other models for their role in assessment of surgical skills.

Cadavers

Cadavers have traditionally been used for medical and surgical anatomy sessions throughout training programs [40]. However, preserved cadaveric tissue does not resemble living tissue and it is not a good model for laparoscopic surgery. The tissues are much stiffer, less malleable, and difficult to work with. Depending on the preserving agent, it is extremely difficult or impossible to create a pneumoperitoneum.  Additionally, cadavers are expensive and in limited supply [44-45].

Animals

Animal models have been used extensively in medical training in the past. There are moral and ethical issues surrounding the use of animal models. Issues such as: is it absolutely necessary to use the animal model, or could these skills be learned in another way? Are the animals undergoing any harm or suffering as a result? Do the benefits to the learner justify the use of the animal? In addition, the high cost and significant resources required to use these animal models have led many universities and programs away from their use [40].

Video trainers      

These refer to the low-fidelity physical box trainers or video trainers. These physical box trainers are composed of frames supporting traditional laparoscopic video monitors, light sources, and camera systems [20]. Trainees use actual laparoscopic instruments to perform tasks under videoscopic guidance [3].

Advantages of the physical box trainers are that they are portable, reusable, and they use the same instruments as used in the operating room [42]. They are also relatively inexpensive with the average cost of a video trainer set up being approximately $2000.

Disadvantages include the fact that they are a low-fidelity technology, the tasks being performed are basic, and it is not possible to perform whole operations. Being able to walk through the steps of an operation helps the trainee to automate the steps and to troubleshoot any difficulties they may encounter in a safe environment [42]. Additionally, an evaluator needs to be present to ensure the tasks are being performed properly and to evaluate the trainee. This limits timing and scheduling and potentially adds an increased cost if the evaluator requires compensation for their time.

Virtual reality simulators  

Virtual reality trainers use computer-generated environments to perform computer-generated tasks. Computer-generated images are linked to a human-computer interface enabling the trainee to manipulate the images and receive objective feedback on performance from the computer [46]. 

There is a spectrum of machines available now, many incorporating haptic feedback. In surgery, haptic or force feedback refers to the sense of touch that a surgeon experiences, both consciously and unconsciously, while operating [47]. Adding haptic feedback to virtual reality simulators used for laparoscopic training and assessment is thought to be beneficial to trainees, especially during the early phase of psychomotor skill acquisition and for complex tasks [47].

Advantages of the virtual reality simulators are that they are reusable, there is no need for an evaluator as there are preset evaluation metrics on the computer, and data can be captured and saved for review at a later time. There is the ability to perform whole procedures, not just technical skills tasks. For example, a trainee can perform a laparoscopic cholecystectomy from start to finish.  In addition, the level of difficulty can be adjusted to create easier or more challenging/stressful situations [17, 42].

Disadvantages of the virtual reality simulators include maintenance, three dimensions are not always well simulated, haptic feedback often lacks realism, and acceptance by trainees is often low [42]. As a result, trainees’ focus and effort on the tasks may be minimal. It then becomes less effective as a training tool and is not an accurate representation of actual laparoscopic skill. They are also extremely expensive compared to the video trainers, with the average cost of a virtual simulator ranging from approximately $80,000 to $120,000. That does not include the maintenance fees, technical support, or the purchase of any additional modules for the machine.

Comparison of video trainers to virtual simulators

Training in lab-based settings with either virtual reality simulators or video trainers results in improvement of skills both in the simulation setting and in the operating room. Simulation is now established as an effective method of skill acquisition [12-23]. 

In the first study to validate the transfer of training skills on a virtual reality simulator to the operating room, Seymour et al. demonstrated improved operative performance during laparoscopic cholecystectomy for residents who had trained on the MIST VR simulator [14]. They showed that residents who had trained on the virtual reality simulator made fewer errors and were faster at completing the cholecystectomy than the control group [14]. They did not include a video training group in their study.

In 2000, Scott et al. demonstrated that 30 minutes of daily video training for 10 days improved video-eye hand skills and translated into improved operative performance for junior surgical residents [21]. Second- and third-year general surgery residents were prospectively randomized to either a video training group or a control group. Both groups were evaluated performing a pre- and posttest laparoscopic cholecystectomy using a validated global operative assessment tool by three evaluators blinded to the resident’s randomization status. All residents were also evaluated performing five standardized video trainer tasks at the beginning and the end of the study. The trained group achieved significantly greater improvement in video trainer scores on all five video trainer tasks, and significantly improved on four out of eight criteria on the global assessment scores for the laparoscopic cholecystectomy [21].

The benefit of one simulation method over the other for training purposes has not been clearly established. Hamilton et al. randomized junior general surgery residents to either a virtual reality or a video trainer structured training program, and assessed their baseline skills compared to their post-training skills [20]. There was no statistically significant improvement difference between the two groups, with the virtual reality group improving their post-test score by 54%, and the video trainer improving theirs by 55%. They also looked at the effect of practice on operative performance by assessing all second-year residents on their operative performance during a laparoscopic cholecystectomy before and after skills training. Operative performance improved only in the virtual reality group (p < 0.05) [20]. 

In another study comparing virtual reality training to video training, Munz et al. randomized 24 novices to a either a control group, video training, or a training on the LapSim virtual reality simulator [13]. The 16 participants assigned to one of the training groups each performed 30 min training sessions once per week under direct supervision. Analysis of pre and post-test scores showed significant improvement in both trained groups, but no significant difference in the improvement between the two groups [13].

A recent Cochrane systematic review concluded that virtual reality training was at least as effective as video training in supplementing standard laparoscopic training, but no clear advantage was demonstrated [15].

Both video trainers and virtual reality simulators have demonstrated significant correlations between operative performance and psychomotor performance in lab-based settings and can be used to assess operative laparoscopic skills [5, 12, 19, 24, 29-30]. There is, however, a significantly larger body of evidence supporting this for the video trainer than the virtual reality simulator, and video trainers are currently used for the technical skills assessment component in the FLS program.  

In 2003, Gallagher et al. demonstrated construct validity of the MIST VR virtual reality simulator, and showed that it was capable of evaluating the psychomotor skills required for the performance of laparoscopic surgery [30].

A study published by Kundhal and Grantcharov in 2008 showed validity of the LapSim virtual reality simulator for assessing laparoscopic skill [19]. They recruited 10 surgical residents with limited laparoscopic experience and had them perform three repetitions of seven basic skills tasks on the LapSim trainer, and then one laparoscopic cholecystectomy in the operating room. Time, error, and economy of motion parameters were measured on the LapSim. Operative performance was video recorded and blindly assessed by two independent observers using a modified OSATS rating scale. The correlation between time, economy of motion, and error parameters during the simulated tasks on the LapSim and the laparoscopic cholecystectomy were statistically assessed. Significant correlations between operative performance and simulator performance were demonstrated [19].

A larger prospective study by Langelotz et al. out of Germany established construct validity of the LapSim virtual reality simulator and showed that it could be used to assess operative laparoscopic skill [29].

There are few studies that directly compare video trainer and virtual reality simulation for assessment of operative laparoscopic skills. In Steigerwald’s study of general surgery residents it was found that construct and predictive forms validity were more thoroughly demonstrated for FLS and the low-fidelity video trainer than the LapVR virtual reality simulator when assessing performance against a live in vivo human cholecystectomy. They concluded there may not be enough evidence to recommend utilizing the LapVR virtual reality simulator for assessment of laparoscopic skills [48]. This is a significant consideration for educators who must balance fiscal responsibility when allocating resources for surgical training.

## Conclusions

Diminishing resources and expanding technologies, such as MAS, have complicated the acquisition and assessment of technical skills in surgical training programs. However, these challenges have been met with both innovation and an evolution in our understanding of how learners develop technical competence and how to better measure it. As these skills continue to grow in breadth and complexity, so too must the surgical education systems ability.
